# Single-cell RNA sequencing of CSF reveals neuroprotective RAC1^+^ NK cells in Parkinson’s disease

**DOI:** 10.3389/fimmu.2022.992505

**Published:** 2022-09-21

**Authors:** Qing Guan, Wei Liu, Ketao Mu, Qi Hu, Jiazhao Xie, Liming Cheng, Xiong Wang

**Affiliations:** ^1^ Department of Laboratory Medicine, Tongji Hospital, Tongji Medical College, Huazhong University of Science and Technology, Wuhan, China; ^2^ Department of Public Health, Tongji Hospital, Tongji Medical College, Huazhong University of Science and Technology, Wuhan, China; ^3^ Department of Radiology, Tongji Hospital, Tongji Medical College, Huazhong University of Science and Technology, Wuhan, China; ^4^ Department of General Medicine, Tongji Hospital, Tongji Medical College, Huazhong University of Science and Technology, Wuhan, China; ^5^ Department of Pathophysiology, Key Laboratory of Ministry of Education for Neurological Disorders, School of Basic Medicine, Tongji Medical College, Huazhong University of Science and Technology, Wuhan, China

**Keywords:** Parkinson’s disease, NK cells, single-cell RNA sequencing, RAC1, brain infiltration

## Abstract

Brain infiltration of the natural killer (NK) cells has been observed in several neurodegenerative disorders, including Parkinson’s disease (PD). In a mouse model of α-synucleinopathy, it has been shown that NK cells help in clearing α-synuclein (α-syn) aggregates. This study aimed to investigate the molecular mechanisms underlying the brain infiltration of NK cells in PD. Immunofluorescence assay was performed using the anti-NKp46 antibody to detect NK cells in the brain of PD model mice. Next, we analyzed the publicly available single-cell RNA sequencing (scRNA-seq) data (GSE141578) of the cerebrospinal fluid (CSF) from patients with PD to characterize the CSF immune landscape in PD. Results showed that NK cells infiltrate the substantia nigra (SN) of 1-methyl-4-phenyl-1,2,3,6-tetrahydropyridine (MPTP)-induced PD model mice and colocalize with dopaminergic neurons and α-syn. Moreover, the ratio of NK cells was found to be increased in the CSF of PD patients. Analysis of the scRNA-seq data revealed that Rac family small GTPase 1 (RAC1) was the most significantly upregulated gene in NK cells from PD patients. Furthermore, genes involved in regulating SN development were enriched in RAC1^+^ NK cells and these cells showed increased brain infiltration in MPTP-induced PD mice. In conclusion, NK cells actively home to the SN of PD model mice and RAC1 might be involved in regulating this process. Moreover, RAC1^+^ NK cells play a neuroprotective role in PD.

## Introduction

Parkinson’s disease (PD) is the second most frequent age-associated neurodegenerative disease following Alzheimer’s disease (AD) and the most common movement disorder. PD is characterized by chronic and selective loss of dopaminergic neurons within the substantia nigra (SN) due to intracellular deposition of misfolded α-synuclein (α-syn) (1). PD affects 1-2% of individuals aged over 60 years, while its prevalence is approximately 5% in individuals over the age of 85 years. More than 10 million people worldwide have PD (2).

Growing evidence suggests that neuroinflammation plays an important role in PD pathogenesis. Neuroinflammation has previously been considered as a local event in the central nervous system (CNS). In PD, neuroinflammation is due to impaired regulation of several chemokines and cytokines secreted by the activated microglia (3). However, perturbations in the peripheral immune system have been reported during early stages of PD (4). The blood-brain-barrier (BBB) prevents the peripheral immune cells from entering the CNS. However, during infection or neurodegenerative conditions, the BBB can be disrupted by free radicals, pro-inflammatory cytokines and chemokines, proteolytic enzymes, and matrix metalloproteinases (5). During PD progression, the integrity of the BBB is compromised, which causes the activated resident innate immune cells within the brain, including microglia and astrocytes, to recruit the peripheral innate immune cells (6). A previous study showed that peripheral lymphocytes can infiltrate the brain *via* the compromised BBB in patients with Lewy body dementia and in MPTP-induced PD model mice (7–9). However, the detailed mechanisms of peripheral innate immune cell infiltration into the CNS remain poorly understood (10).

Herein, we analyzed the publicly available single-cell RNA sequencing (scRNA-seq) data (GSE141578) of the cerebrospinal fluid (CSF) cells from patients with PD to characterize the CSF immune signature in these patients. Moreover, combined with the immunofluorescence staining of the SN region of 1-methyl-4-phenyl-1,2,3,6-tetrahydropyridine (MPTP)-induced PD mouse model, our study revealed the neuroprotective role of RAC1-positive natural killer (NK) cells in PD.

## Methods

### MPTP-induced PD model mice

Adult male C57BL/6 mice were purchased from Beijing Vital River Laboratory Animal Technology Company (Beijing, China) and housed in a temperature-controlled room (22-24 °C) under a 12-12 h light-dark cycle. MPTP (Cat No. S4732) and probenecid (Cat No. S4022) were purchased from Selleck (Shanghai, China). For the development of chronic MPTP PD model, mice were injected subcutaneously with 25 mg/kg MPTP and intraperitoneally with 250 mg/kg probenecid every 3.5 days for 10 times (11). All experimental procedures were approved by the Committee of Tongji Hospital, Tongji Medical College, Huazhong University of Science and Technology.

### Immunofluorescence staining

The brains were prefixed by perfusion with 4% paraformaldehyde and sectioned using the Leica freezing microtome. The sections (25 μm thick) were rinsed with PBS and incubated with 0.5% Triton X-100 in PBS for 30 min for blocking. Following this, the brain sections were incubated with primary antibodies overnight at 4 °C, followed by incubation with Alexa Fluor 488 goat anti-rabbit or Alexa Fluor 594 goat anti-mouse secondary antibody for 1 h in the dark at room temperature. To stain the DNA, sections were incubated with DAPI for 5 min. Stained sections were imaged using a scanning microscope (SV120, Olympus, Japan). Rabbit polyclonal NKp46 (Cat No.: PA5-102860; Invitrogen, CA, USA), mouse monoclonal RAC1 (Cat No.:66122-1-Ig; Proteintech, Wuhan, China), mouse monoclonal tyrosine hydroxylase (Cat No.: TA506541; OriGene Technologies, Wuxi, China), and mouse monoclonal α-syn (Cat No.: BS-0012M; Bioss, MA, USA) antibodies were used.

### Single-cell RNA sequencing data analysis

The publicly available scRNA-seq data (GSE141578) of the CSF cells from PD patients were downloaded and analyzed. Cells in the CSF were sorted using the LSRFortessa cell analyzer (BD Biosciences, CA, USA). RNA was purified and library was prepared according to the manufacturer's instructions using the 10x v2 5' Expression library kit. The software RTA2 was used for basecalling, and fastq files were generated using Cellranger mkfastq (v3.0.2), followed by alignment to the GRCh38 3.0.0 reference genome available at 10X Genomics using Cellranger. Expression matrices generated using the cellranger were loaded into the Seurat (v4.0.2) R package for downstream uniform manifold approximation and projection (UMAP) analysis (12). Cells with <500 genes, <3% ribosome genes, >0.1% hemoglobin genes, or >50% mitochondrial genes were excluded from the analysis. A total of 18,553 CSF cells from 14 subjects (seven PD patients and seven healthy controls) were selected for analysis. The expression matrix was normalized using the LogNormalization method and further scaled using all genes. Two thousand highly variable genes were selected using the vst method. The Harmony (v0.1.0) R package was used to correct for the batch effect of sample identity (13). Principal component analysis (PCA) was performed to identify significant principal components (PCs), and 15 PCs were used for UMAP and FindNeighbors analyses. The FindClusters function was used to classify the cells into 16 different clusters with a resolution of 0.8. The FindAllMarkers method with default parameters was used to identify differentially expressed genes (DEGs) in each cluster. The cell type was identified based on DEGs and manually checked according to a previously published study (14). The FindMarkers method with default parameters was used to identify DEGs between patients with PD and healthy controls within each cell type.

### Transcription factor activity analysis

DoRothEA is a gene regulatory network that contains transcription factor (TF)-target gene interactions (15). Human DoRothEA regulons with confidence levels A, B, or C based on supporting evidence were used. The Viper scores were calculated using the scale method. The top ten most variable TFs were identified and plotted.

### Gene ontology enrichment analysis

DEGs between RAC1_high and RAC1_low NK cells were identified using the FindMarker method in Seurat. The upregulated genes in RAC1 high NK cells were used as input for Gene ontology (GO) enrichment analysis using the clusterProfiler (v4.0) R package, and the top five GO terms were plotted (16).

## Results

### NK cells colocalize with α-synuclein deposits in the SN of MPTP-induced PD model mice

**Figure 1:**
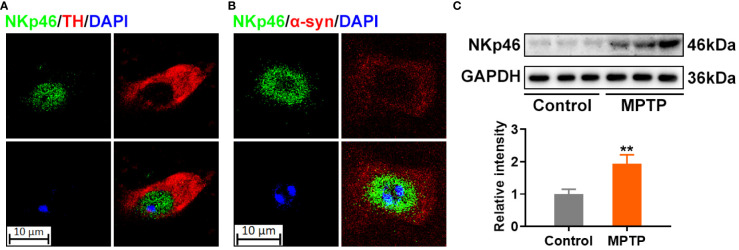
NK cells colocalize with α-synuclein deposits in the SN of MPTP-induced PD model mice. **(A)** NK cells were stained using NKp46 antibody in green, and dopaminergic neurons were labelled by TH in red in the SN of MPTP-induced PD mice. **(B)** NK cells were stained using NKp46 antibody in green, and α-syn was labelled by TH in red in the SN of MPTP-induced PD mice. DNA was stained using DAPI. The results implicate that NK cells reside with α-syn deposits, and colocalized with dopaminergic neurons in the SN of MPTP-induced PD brain. **(C)** Protein level of NK marker was detected using NKp46 antibody *via* western blotting in the SN region of MPTP-induced PD and control mice. Increased NK cells were found as revealed by increased NKp46 level. **p<0.01 compared with control group.

NK cells are present in the SN of patients with PD and colocalize with the preformed fibrils (PFF) in α-syn PFF-injected mice model (17). The MPTP-induced mouse model is a gold standard in PD research for decades (18). Our immunofluorescence assay showed that NK cells colocalize with the tyrosine hydroxylase (TH)-positive dopaminergic neurons in the SN (**Figure 1A**) and α-syn deposits in the brains of MPTP-induced PD model mice (**Figure 1B**). Moreover, the expression of the NK cell marker NKp46 was increased in the SN region of MPTP-induced PD model mice (**Figure 1C**).

### The ratio of NK cells is increased in the CSF of PD patients

**Figure 2:**
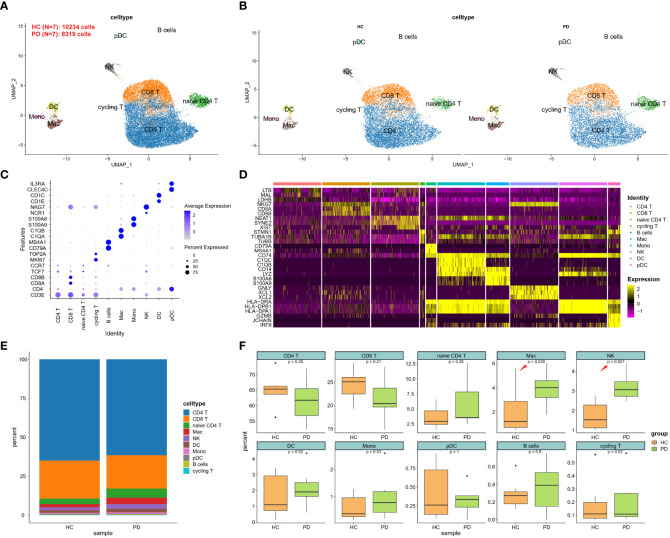
scRNA-seq analysis of CSF cells in PD. **(A)** UMAP projection of 18,553 cells from all CSF cells. **(B)** UMAP projection of PD and HC groups, respectively. NK cells were labeled with ellipse tag, which were decreased in PD group. **(C)** Dot plot of cell type marker genes. CD4 T was marked using CD3 epsilon subunit of T-cell receptor complex (CD3E) and CD4 molecule (CD4); CD8 T was marked using CD3E, CD8a molecule (CD8A), and CD8b molecule (CD8B); naïve CD4 T was marked using CD3E, CD4, transcription factor 7 (TCF7), and C-C motif chemokine receptor 7 (CCR7); cycling T was marked CD3E, marker of proliferation Ki-67 (MKI67), and DNA topoisomerase II alpha (TOP2A); B cell was marked using CD79a molecule (CD79A), and membrane spanning 4-domains A1 (MS4A1); Macrophage (Mac) was marked using complement C1q A chain (C1QA), and complement C1q B chain (C1QB); Monocyte (Mono) was marked using S100 calcium binding protein A9 (S100A9), and S100 calcium binding protein A8 (S100A8); Natural killer (NK) was marked using natural cytotoxicity triggering receptor 1 (NCR1), and natural killer cell granule protein 7 (NKG7); Dendritic cell (DC) was marked using CD1e molecule (CD1E), and CD1c molecule (CD1C); Plasmacytoid dendritic cell (pDC) was marked using C-type lectin domain family 4 member C (CLEC4C), and interleukin 3 receptor subunit alpha (IL3RA). **(D)** Heatmap of top 3 DEGs in each cell type. These cell types could be well classified using these cell-type specific DEGs. **(E)** Cell type distribution in each group. **(F)** Cell type distribution in PD and HC group. The percent of Mac and NK was decreased in PD CSF.

Dysfunctional BBB in PD may lead to the passive entry of NK cells into the brain. To uncover the potential mechanisms of NK cell homing to the brain, we analyzed the scRNA-seq data (GSE141578) from the publicly available GEO database provided by Gate D et al., (7). A total of 8,319 cells were isolated from the CSF of patients with PD (n = 7), while 10,234 cells were isolated from healthy controls (HC, n = 7) (**Table 1**). These cells were assigned to known cell types based on the expression of specific marker genes (19). The 16 identified cell clusters were visualized using the UMAP analysis (**Figure 2A** and **Figure S1**). Clusters were not specific to any group (**Figure 2B**) and expressed marker genes corresponding to each subtype (**Figures 2C-D** and **Table S1**). Based on DEGs, the majority of cells were found to be CD4^+^ and CD8^+^ T cells (**Figure 2E**). Furthermore, the ratio of NK cells and macrophages (Mac) was found to be higher in the PD group (**Figure 2F**).

**Table 1:** A total of 18,553 CSF cells were isolated from 14 subjects.

Sample	Cells	Group
GSM4208772	1320	HC
GSM4208773	1530	HC
GSM4208774	1836	HC
GSM4208775	2000	HC
GSM4208778	1305	HC
GSM4208779	1423	HC
GSM4404055	820	HC
GSM4208766	1433	PD
GSM4208768	524	PD
GSM4208769	1195	PD
GSM4208770	1068	PD
GSM4404054	769	PD
GSM4404056	1474	PD
GSM4404057	1856	PD

### RAC1^+^ NK cells are associated with SN development

**Figure 3:**
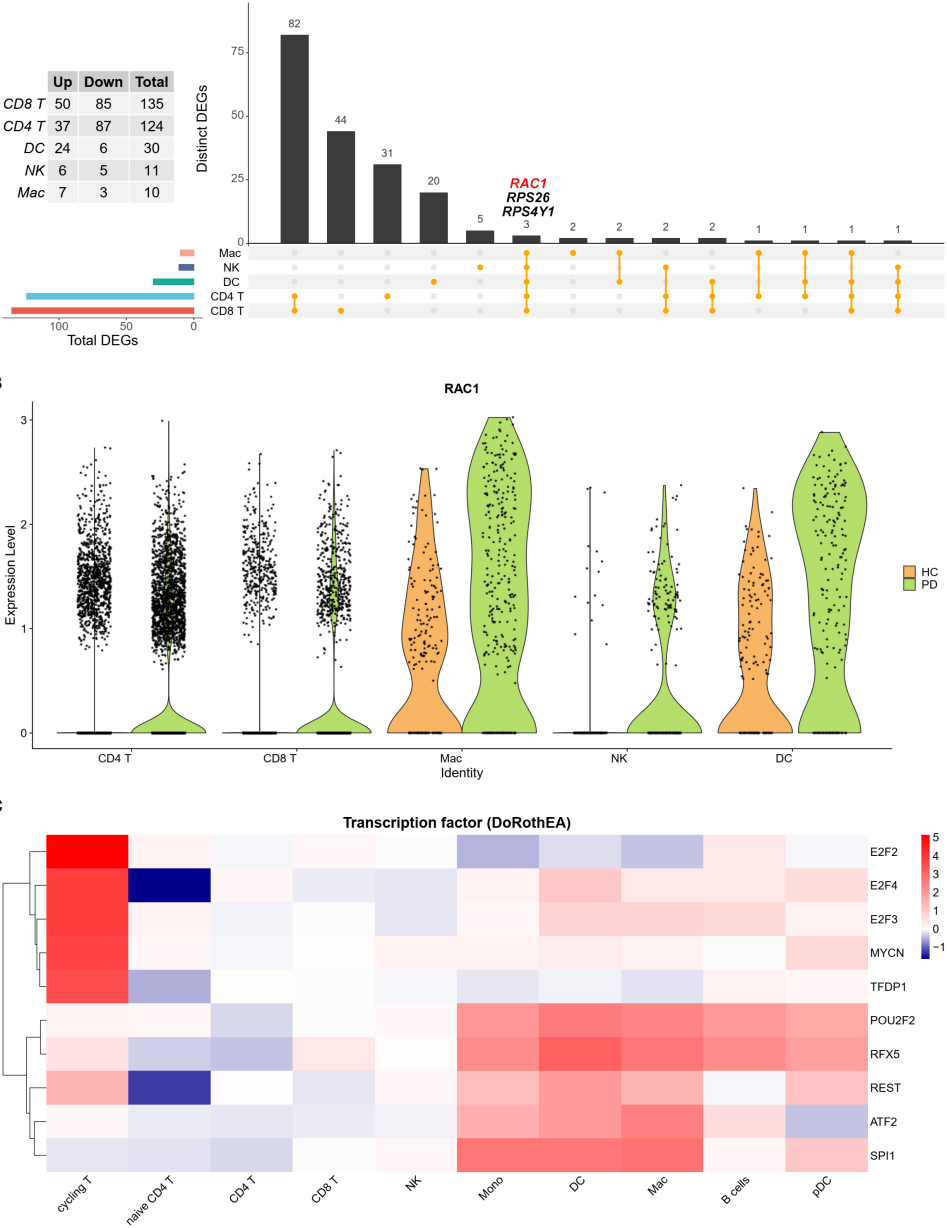
The ratio of NK cells is increased in the CSF of PD patients. **(A)** Upset plot showed the intersection of DEGs among five main cell types. RAC1 was increased in PD group in all five cell types. **(B)** Violin plot depicts distributions of RAC1 in each cell type between PD and HC group. HC group was colored in orange and PD group was colored in green. RAC1 was increased in PD group across five main cell types. **(C)** TF activity was calculated using DoRothEA R package, and the top 10 most variable TFs were plotted. Cycling T cells showed the most active TF activity, while naïve T cells showed the least TF activity.

Analysis of the cell clusters revealed that CD8^+^ T cells were the most transcriptionally dysregulated immune cell subtype. Moreover, Rac family small GTPase 1 (RAC1), a small GTP-binding protein essential for cell migration and invasion, was differentially expressed in all five cell types, including CD8^+^ T cells, CD4^+^ T cell, dendritic cells (DCs), NK, and Mac (**Figure 3A**; **Table S2**). These five cell types accounted for 93.2% and 91.7% of all cells in HC and PD groups, respectively. Compared to the HC group, the expression of RAC1 was found to be higher in all five main cell types in the PD group. Moreover, fewer NK cells were RAC1 positive in the HC group compared to the PD group (**Figure 3B**). TF activity was analyzed in each cell type using the DoRothEA R package. Results showed that cycling and naïve T cells had the highest and lowest TF activity, respectively (**Figure 3C**). Low TF activity was also observed in NK cells, indicating that post-transcriptional modifications might contribute to NK-specific differential gene expression.

**Figure 4:**
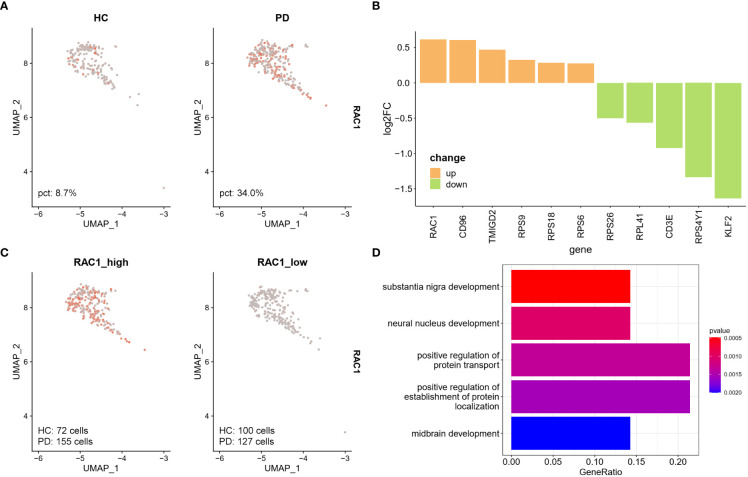
RAC1^+^ NK cells are associated with SN development. **(A)** Feature plot of RAC1 in NK cells. PD group showed increased RAC1 expression. **(B)** DEGs between PD and HC group in CSF NK cells. Upregulated DEGs were colored in orange, and downregulated DEGs were colored in green. RAC1, Rac family small GTPase 1; CD96, CD96 molecule; TMIGD2, transmembrane and immunoglobulin domain containing 2; RPS9, ribosomal protein S9; RPS18, ribosomal protein S18; RPS6, ribosomal protein S6; RPS26, ribosomal protein S26; RPL41, ribosomal protein S41; CD3E, CD3 epsilon subunit of T-cell receptor complex; RPS4Y1, ribosomal protein S4 Y-linked 1; KLF2, KLF transcription factor 2. **(C)** The NK cells were divided into RAC1_high and RAC1_low groups according to the median expression level of RAC1 calculated by AddModuleScore function in Seurat. 55% of NK cells from PD group were RAC1_high, while 42% of NK cells from HC were RAC1_high. **(D)** Upregulated DEGs in RAC1_high compared with RAC1_low group were enriched in SN development, neural nucleus development, and midbrain development biological process GO terms.

The UMAP plot showed that the NK cell-specific expression of RAC1 was lower in the HC group compared to the PD group (**Figure 4A**), and RAC1 was the top most differentially expressed gene in NK cells (**Figure 4B**). NK cells were divided into two groups (RAC1_high and RAC1_low) according to the median expression level of RAC1 calculated using the AddModuleScore function in Seurat. Results showed that 55% of NK cells from the PD group were RAC1_high, while 42% of them from the HC group were RAC1_high (**Figure 4C**). Genes differentially expressed between the RAC1_high and RAC1_low NK cells were enriched in SN development, neural nucleus development, and midbrain development (**Figure 4D**; **Table S3**). Thus, RAC1^+^ NK cells in the CSF can be considered as neuroprotective.

### RAC1^+^ NK cells home to the SN of MPTP-induced PD model mice

**Figure 5:**
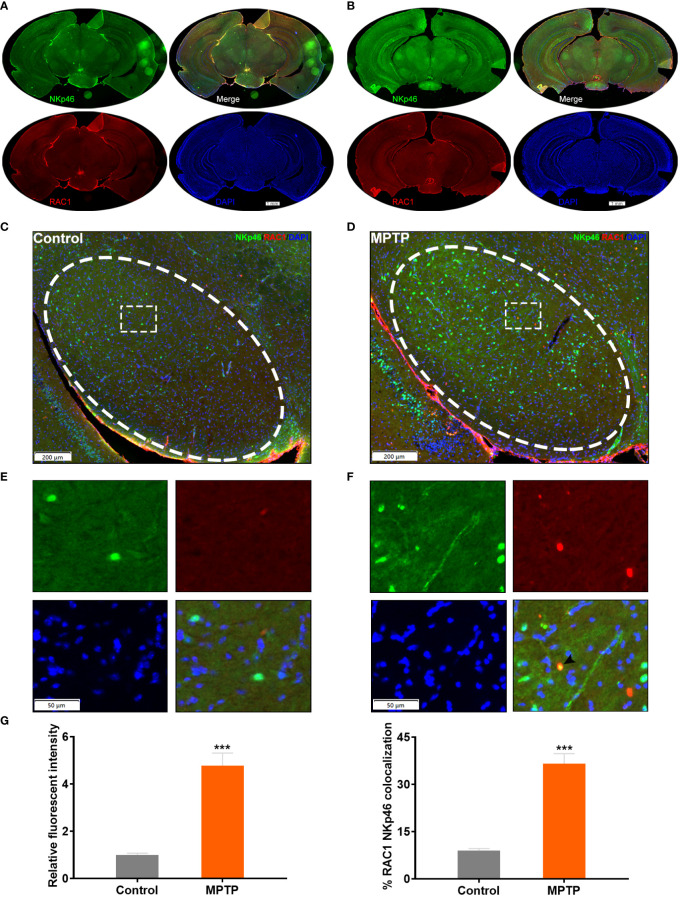
RAC1^+^ NK cells home to the SN of MPTP-induced PD model mice. **(A)** Immunofluorescence staining of NKp46 (green) and RAC1 (red) in the brain of control mice. Whole slice was shown. **(B)** Immunofluorescence staining of NKp46 (green) and RAC1 (red) in the brain of MPTP-induced PD mice. Whole slice was shown. **(C)** Immunofluorescence staining image of the SN region (labeled with ellipse tag) in control mice. **(D)** Enlarged image of the dashed rectangle in **(C, E)** Immunofluorescence staining image of the SN region (labeled with ellipse tag) in PD mice. **(F)** Enlarged image of the dashed rectangle in **(E)** Arrowhead showed the merged staining of NKp46 and RAC1. **(G)** The relative fluorescent intensity of NKp46 and RAC1/NKp46 colocalization were calculated. Increased NKp46 level and RAC1/NKp46 colocalization were observed in MPTP group. ***p<0.001 compared with control group.

Next, we examined the SN of the MPTP-induced PD model mice to localize and quantify RAC1^+^ NK cells (**Figures 5A, B**). Immunofluorescence analysis showed increased number of RAC1^+^ NK cells co-expressing NKp46 in the SN of PD mice (**Figures 5C-G**). These results support the hypothesis that RAC1^+^ NK cells home to the SN in PD model mice.

## Discussion

Increasing evidence suggests that the peripheral immune system plays an essential role in the regulation of CNS homeostasis and diseases (20). Brain infiltration of the NK cells has been widely observed in neurodegenerative disorders, including AD and PD, and may play diverse roles under different conditions (14, 17). In a triple transgenic mouse model of AD (3xTg-AD), NK cells exhibit an enhanced pro-inflammatory profile, and antibody-mediated depletion of NK cells in these transgenic mice improves cognitive functions by enhancing neurogenesis and reducing neuroinflammation without affecting the levels of amyloid beta (21). Recent studies showed that the number of NK cells is higher in the peripheral blood and CNS of patients with PD. NK cells can internalize α-syn aggregates, and systemic depletion of NK cells exacerbates synuclein pathology in the α-syn PFF-injected mouse model of PD, suggesting a neuroprotective role of NK cells (17, 22). These studies indicate differential roles of NK cells under different pathogenic conditions. We previously showed that STAT3 regulates the expression of immunity-related genes in peripheral NK cells and promotes the brain infiltration of NK cells and neuroinflammatory changes in AD (14). However, the detailed mechanisms underlying brain infiltration of NK cells in PD remain unclear.

The BBB consists of specialized microvascular endothelial cells surrounded by mature pericytes and outer astrocytic end-feet (23). The integrity of the BBB is critical for angiogenesis, normal neuronal functions, and synaptic remodeling. BBB disruption has been observed in several neurodegenerative disorders including PD (24). α-syn can be bidirectionally transported between the brain and peripheral circulation, and oligomeric α-syn causes BBB disruption leading to increased BBB permeability, which is mediated by astrocyte-derived vascular endothelial growth factor A and nitric oxide (25). The peripheral immune cells migrate to the CSF *via* the disrupted BBB. The immune signature of the CSF has helped in understanding the potential mechanisms of peripheral CD4^+^ T cell homing to the brain and its contribution to neurodegeneration in Lewy body dementia (8).

Our study confirms the homing of NK cells to the brain in a mice model of PD. The co-localization of NK cell markers and TH, a marker of dopaminergic neurons, as well as α-syn in the SN region indicates the involvement of NK cells in local CNS neuroinflammation. Our results are consistent with those of a previous study showing the phagocytic action of NK cells on α-syn (17). The scRNA-seq data analysis further showed the potential pathways involved in regulating the NK cell infiltration into the brain. The ratio of NK cells and macrophages was found to be higher in the CSF of PD patients, and this change might be associated with altered immune functions. The analysis of DEGs between HC and PD groups showed that RAC1 is upregulated in all five cell types analyzed. Moreover, RAC1 was the most upregulated gene in NK cells when comparing PD vs HC groups. Our TF activity analysis partially excluded the transcriptional regulation of RAC1, as revealed by the low TF activity in NK cells. The function of RAC1^+^ NK cells was analyzed using the GO enrichment analysis, and results showed that the GO terms, including SN development, neural nucleus development, and midbrain development, were significantly enriched in RAC1_high NK cells, strongly suggesting a neuroprotective role of RAC1_high NK cells. Co-localization of RAC1 and the NK cell marker NKp46 further confirmed the presence of RAC1^+^ NK cells in the SN of PD mice brains. Our combined analysis of MPTP-induced PD mice and scRNA-seq data of CSF from PD patients demonstrated the neuroprotective role of RAC1^+^ NK cells in PD.

RAC1 is a small GTPase that plays a critical role in regulating cytoskeletal dynamics. In migrating cells, RAC1 promotes lamellipodia formation by triggering actin polymerization (26). RAC1 inhibition using NSC23766 prevents the NK cell migration following lenalidomide stimulation, while RAC1 activation is mediated by the protein cereblon (27). NK cells expressing dominant-negative RAC1 are less cytotoxic with compromised migration abilities (28). RAC1 induces the proinflammatory NK cells by driving membrane dynamics (29). RAC1 activation contributes to HIV-1 induced monocyte-BBB interactions and HIV-1 infection of the brain macrophages (30). RAC1 promotes CD4^+^ T cell migration in arteriosclerosis obliterans (31). In our scRNA-seq data analysis, RAC1 expression was found to be increased in the five main cell types. Moreover, RAC1 expression was found to be significantly higher in NK cells from PD patients compared to those from health controls. Therefore, we speculate that RAC1 might be partially involved in regulating the migration of peripheral immune cells from the blood to the CSF.

In conclusion, our study confirms that NK cells home to the SN of MPTP-induced PD mice. Increased NK cell ratio in the CSF of patients with PD might be partially mediated by RAC1. Finally, RAC1 might be involved in regulating the brain infiltration of NK cells and RAC1^+^ NK cells play a neuroprotective role in PD.

## Data availability statement

Publicly available datasets were analyzed in this study, the name of the repository and accession number can be found below: NCBI under accession ID: GSE141578.

## Ethics statement

The animal study was reviewed and approved by the Committee of Tongji Hospital, Tongji Medical College, Huazhong University of Science and Technology.

## Author contributions

QG, LC, and XW conceived and designed the study; WL, QH, KM, and JX collected data; XW wrote the paper. All authors contributed to the article and approved the submitted version.

## Funding

This work was supported by grants from the National Natural Science Foundation of China (No. 81500925 to XW, 81901713 to KM). This work was supported by the Tongji Hospital (HUST) Foundation for Excellent Young Scientist (Grant No. 2020YQ01-11 to XW).

## Acknowledgments

We thank Dr. Jianming Zeng (University of Macau), and all the members of his bioinformatics team, biotrainee, for generously sharing their experience and codes. The Use of the biorstudio high performance computing cluster (https://biorstudio.cloud) at Biotrainee and The shanghai HS Biotech Co.,Ltd for conducting the research reported in this paper.

## Conflict of interest

The authors declare that the research was conducted in the absence of any commercial or financial relationships that could be construed as a potential conflict of interest.

## Publisher’s note

All claims expressed in this article are solely those of the authors and do not necessarily represent those of their affiliated organizations, or those of the publisher, the editors and the reviewers. Any product that may be evaluated in this article, or claim that may be made by its manufacturer, is not guaranteed or endorsed by the publisher.

## Abbreviations

PD, Parkinson’s disease; AD, Alzheimer’s disease; SN, substantia nigra; α-syn, α-synuclein; CNS, central nervous system; BBB, blood-brain-barrier; scRNAseq, single-cell RNA sequencing; CSF, cerebrospinal fluid; MPTP, 1-methyl-4-phenyl-1,2,3,6-tetrahydropyridine; UMAP, Uniform Manifold Approximation and Projection; PCA, Principal component analysis; PC, principal components; DEGs, differentially expressed genes; HC, healthy control; TF, transcription factor; GO, Gene ontology; PFF α-syn, fibrils of α-syn; TH, tyrosine hydroxylase; RAC1, Rac family small GTPase 1.
