# Supplementary figures and images for: Single-cell RNA sequencing of CSF reveals neuroprotective RAC1+ NK cells in Parkinson’s disease
Source: Front Immunol. 2022 Sep 21;13:992505. doi: 10.3389/fimmu.2022.992505 (PMC9532252; doi:10.3389/fimmu.2022.992505)

# RNA\_snn\_res.0.8

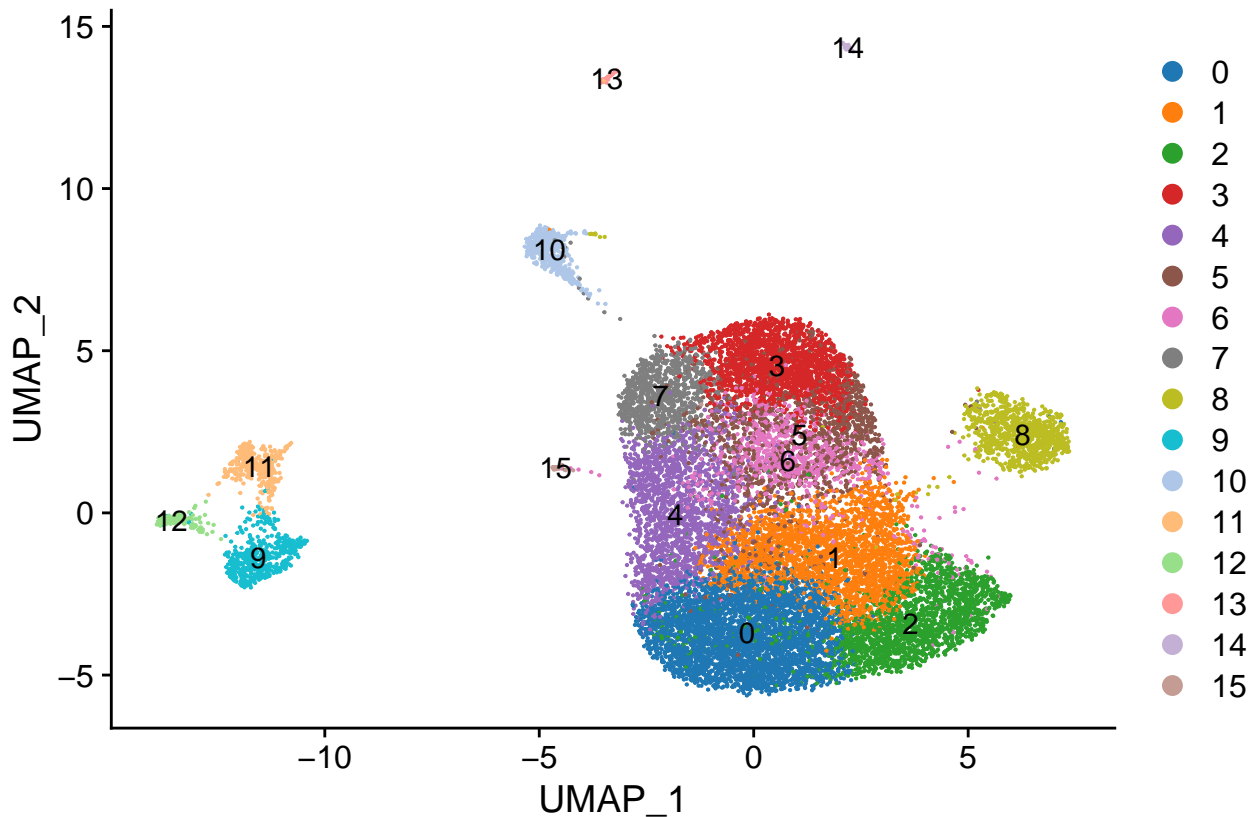

Supplement: Supplementary file 1 [file DataSheet_1.pdf]
